# A systematic review and meta-analysis of randomised controlled trials of peer support for people with severe mental illness

**DOI:** 10.1186/1471-244X-14-39

**Published:** 2014-02-14

**Authors:** Brynmor Lloyd-Evans, Evan Mayo-Wilson, Bronwyn Harrison, Hannah Istead, Ellie Brown, Stephen Pilling, Sonia Johnson, Tim Kendall

**Affiliations:** 1 Mental Health Sciences Unit, University College London, Charles Bell House, 67-73 Riding House Street, London W1W 7EJ, UK; 2 Centre for Outcomes Research and Effectiveness, Research Department of Clinical, Educational & Health Psychology, University College London, 1-19 Torrington Place, London WC1E 7HB, UK; 3 University of the West of England, Glenside Campus, Blackberry Hill, Bristol BS16 1DD, UK; 4 Sheffield Health & Social Care NHS Foundation Trust, Fulwood House, Sheffield S10 3TH, UK

**Keywords:** Psychiatry and psychology, Mental health services, Schizophrenia and disorders with psychotic features

## Abstract

**Background:**

Little is known about whether peer support improves outcomes for people with severe mental illness.

**Method:**

A systematic review and meta-analysis was conducted. Cochrane CENTRAL Register, Medline, Embase, PsycINFO, and CINAHL were searched to July 2013 without restriction by publication status. Randomised trials of non-residential peer support interventions were included. Trial interventions were categorised and analysed separately as: mutual peer support, peer support services, or peer delivered mental health services. Meta-analyses were performed where possible, and studies were assessed for bias and the quality of evidence described.

**Results:**

Eighteen trials including 5597 participants were included. These comprised four trials of mutual support programmes, eleven trials of peer support services, and three trials of peer-delivered services. There was substantial variation between trials in participants’ characteristics and programme content. Outcomes were incompletely reported; there was high risk of bias. From small numbers of studies in the analyses it was possible to conduct, there was little or no evidence that peer support was associated with positive effects on hospitalisation, overall symptoms or satisfaction with services. There was some evidence that peer support was associated with positive effects on measures of hope, recovery and empowerment at and beyond the end of the intervention, although this was not consistent within or across different types of peer support.

**Conclusions:**

Despite the promotion and uptake of peer support internationally, there is little evidence from current trials about the effects of peer support for people with severe mental illness. Although there are few positive findings, this review has important implications for policy and practice: current evidence does not support recommendations or mandatory requirements from policy makers for mental health services to provide peer support programmes. Further peer support programmes should be implemented within the context of high quality research projects wherever possible. Deficiencies in the conduct and reporting of existing trials exemplify difficulties in the evaluation of complex interventions.

## Background

Peer support includes support or services provided to people with mental health problems by other people who have experienced mental health problems themselves [1]. Organised peer support is designed to build upon naturally occurring support among people with mental health problems. Peer support has been proposed as a way to promote recovery for anyone who has experienced mental ill health, irrespective of diagnosis [2]. For example, it may promote self-efficacy and hope through sharing experiential knowledge and through modelling recovery and coping strategies [3]. This is consistent with psychological theories of change: peers’ social proximity to the people they are supporting may enhance their value as pro-social models [4] and promote motivation to achieve recovery by providing an upward social comparison [5]. The potential for recipients of peer support to also provide reciprocal support, explicit in mutual support groups and implicit in peer relationships generally, may be empowering and of therapeutic value. Peer support workers may also be able to deliver specific interventions that could be provided by clinicians. However, peer support is explicitly *not* based on psychiatric models of illness [2], and peer support programmes may not be highly specified or theory-driven. In some programmes, mechanisms of action and anticipated outcomes are not clearly described.

Access to peer support for people with severe mental health problems has been widely advocated internationally by service user researchers [6-8] and by professional organisations [9-11]. Provision of peer support is identified as a fidelity requirement for recovery-orientated services [12], and it is commonly promoted in recovery literature [13,14]. The provision of peer support as part of community mental health services is increasingly common. Peer support is now reimbursable in 27 states in the US [15]. In the UK, employment of peer support workers within state mental health services was rare before 2010, but some NHS Trusts now employ in excess of 20 peer support workers [16].

There have been at least nine reviews of peer support; however, these include narrative reviews, [1,17-19] reviews limited to sub-types of peer support [20-22], and a review limited to peer support for people with depression only [23]. Another systematic review of all types of peer support is now a decade old [24]. For these reasons, a current systematic review of available evidence from all randomised controlled trials of organised, community-based peer support for people with severe mental illness is required in order to describe the content of different interventions, organise them according to the best available typologies, synthesise their outcomes, and describe the quality of existing evidence.

### Aims of the study

This paper systematically reviews trials of community-based, peer-provided support for people with severe mental illness. Peer support has been organised in three pre-defined subgroups, which are theoretically distinct [13,17], and which include comparators that would be inappropriate to combine (e.g. in meta-analysis).

i) *Mutual support groups* in which relationships are thought to be reciprocal in nature, even if some participants are viewed as more experienced or skilled than others;

ii) *Peer-support services* in which support is primarily uni-directional, with one or more clearly defined peer supporters offering support to one or more programme participants (support is separate from or additional to standard care provided by mental health services);

iii) *Peer mental health service providers*: people who have used mental health services and are employed to provide part or all of the standard care delivered by a mental health care service (i.e. the difference from standard care should be the provider rather than the role).

Peer mental health providers are thus explicitly aiming to provide services which are also be provided by clinicians; the content of mutual support groups is largely unspecified and peer support per se is the intervention; peer support services are designed as a peer-provided addition to standard care.

## Methods

We evaluated the effects of peer-provided interventions on objective outcomes including hospitalisation and employment, and on self-reported outcomes including symptoms of mental health problems, quality of life, recovery, hope, empowerment, and satisfaction with services. The review protocol was pre-specified [25]. This review of previously reported studies required no ethical approval or additional consent from participants.

### Eligibility criteria

#### ***Types of trials***

Randomised controlled trials (RCTs) including cluster RCTs and factorial RCTs were included. Published and unpublished trials were eligible.

#### ***Types of participants***

Studies were included if participants were adults with severe mental illness. We included participants with schizophrenia spectrum or bipolar disorder, or studies with mixed populations of people using secondary mental health services. We excluded studies including only participants with unipolar depression or personality disorders. Peers were using or had used secondary mental health services.

#### ***Types of interventions***

Included interventions were community-based peer support designed to facilitate recovery from severe mental illness. We included studies of peer support in addition to other interventions if the effect of peer support could be isolated. We excluded: residential and inpatient peer-run programmes; peer support programmes focusing exclusively on areas other than overall mental health recovery (e.g. employment, physical health or drug and alcohol use); and interventions led by mental health professionals.

When studies included more than one eligible intervention, we combined them for analysis. When studies included multiple comparison groups, we included all comparisons that allowed us to isolate the effects of peer-provided interventions (e.g. peer support with treatment as usual (TAU) compared with TAU) or that directly compared peer support with another intervention (e.g. peer-led versus professionally-led services). Other groups were not analysed (see Additional file 1).

### Types of outcome measures

1) Hospitalisation

2) Employment

3) Overall psychiatric symptoms

4) Symptoms of psychosis

5) Depression and anxiety

6) Quality of Life

7) Recovery (self-rated)

8) Hope

9) Empowerment

10) Satisfaction with services

### Search strategy

We searched the Cochrane Central Register of Controlled Trials (CENTRAL), CINAHL, Embase, Medline, preMedline, and PsycINFO from inception to January 2013, combining synonyms for: severe mental illness; peer support; and randomised controlled trial, using the AND command (see Additional file 1). The search was updated in July 2013 as part of a broader search for trials of interventions for psychosis. Reference lists of reviews, included studies, and excluded studies were searched for additional citations. We contacted study authors and other experts. Two authors independently screened abstracts (BH and HI) and resolved differences with a third author (BLE).

### Assessment of bias

Studies were assessed using the Cochrane Collaboration Risk of Bias Tool. Two authors rated each study for risk of bias due to: sequence generation; allocation concealment; blinding of participants, assessors and providers; selective outcome reporting; and incomplete data. Risk of bias for each domain was rated as high (seriously weakens confidence in the results), low (unlikely to seriously alter the results) or unclear. Discrepancies were resolved through discussion.

### Data management

Data were extracted independently by two reviewers (HI or BLE, and BH). We extracted data regarding outcomes at all-time points, study characteristics (setting, number randomised, duration), inclusion criteria and participant demographics, and characteristics of the interventions. We also contacted all authors to request missing data on participant characteristics and outcomes, and to enquire about unpublished studies.

### Statistical analysis

For continuous outcomes, we calculated the standardised mean difference, Hedges *g,* and weighted studies using the inverse of variance. For dichotomous outcomes, we calculated risk ratios (RR) and combined studies using the Mantel-Haenszel method. All outcomes are reported with 95% confidence intervals (CI) using random-effects models.

Missing data were noted for each outcome. When dropout was not reported, we contacted the authors. When analyses were reported for completers as well as controlling for dropout (for example, imputed using regression methods), we used the latter.

Statistical heterogeneity was assessed by visual inspection of forest plots, by performing the Chi^2^ test (assessing the P value) and by calculating the I^2^ statistic, which describes the percentage of observed heterogeneity that would *not* be expected by chance. If the p value was less than 0.10 and I^2^ exceeded 50%, we considered heterogeneity to be substantial. When subgroup analyses were conducted, differences between groups were tested using Chi^2^. Meta-analysis was conducted using RevMan and a summary of results was prepared using the GRADE system [26], which is a structured assessment of confidence in the evidence for individual outcomes attending: threats to internal validity, inconsistency, indirectness, imprecision, and reporting bias. For example, results with I^2^ > 50% were downgraded for study quality. The GRADE system rates confidence in the evidence from each analysis of pooled data as high, moderate, low or very low.

## Results

### Trial flow

**Figure 1:**
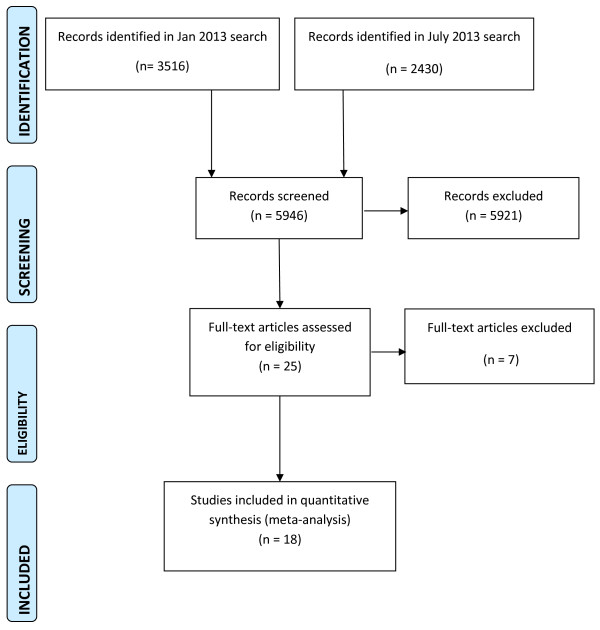
PRISMA flowchart of review search.

We conducted a specific search for peer support interventions on January 2013 which identified 3516 citations. The search was updated on July 2013 as part of a broader search for trials of interventions for psychosis, identifying a further 2430 citations. Together, the searches identified 5946 citations. Papers were excluded following abstract screening if they evidently evaluated interventions other than peer support or did not involve a severely mentally ill population. Twenty-five full-text articles were considered, and 18 trials were included (Figure 1). Sixteen studies reported data that could be included in a meta-analysis; two provided no usable data [27,28]. Seven trials were excluded because the intervention was mainly clinician-led, despite being described as peer support; [29-31] the intervention targeted only clinical or social needs not specific to severe mental illness (e.g. physical health; drug and alcohol use; employment); [32-34] or the intervention involved additional clinical care and peer support, so the independent effect of peer support could not be estimated [35].

### Study characteristics

Trials involved 5597 participants with a median sample size of 178, ranging from 33 to 1827. The 16 trials that were analysed included 5383 participants (96% of people included in the review). Seventeen trials were individually randomised and one was a cluster randomised trial [36] that assigned six services to intervention or control. In this cluster-randomised trial, only 57% of service users within the services randomised to the intervention group accessed the peer support service.

In all studies of mutual support (4) and peer-support (11), interventions were provided in addition to TAU and compared to TAU alone (Table 1). Three studies examined peer-delivered mental health services compared to TAU.

**Table 1:** Characteristics of included studies

**Study**	**Location**	**No.**	**Diagnosis**^**Φ**^	**Age (yr)**	**Sex (F)**	**Race (BME)**	**Employment**^**Ω**^
*Mutual Support*							
Edmundson 1982 [51]	Florida *USA*	80	N/R	N/R	N/R	N/R	N/R
Kaplan 2011 [47]^ψ^	Pennsylvania *USA*	300	22% SS 78% mood	47	66%	13%	64%
Rogers 2007 [42]	Multiple sites *USA*	1827	50% SS 44% mood	43	60%	43%	29%
Segal 2011 [49]	California *USA*	162	41% SS 59% mood	37	54%	13%	4%
*Peer Support*							
Barbic 2009 [39]	Ontario *CA*	33	79% SS 21% BPD	45	33%	N/R	12%
Chinman 2013 [36]	South Western USA	282^α^		53	11%	46%	N/R
Cook 2012 [46]	Tennessee, *USA*	428	21% SS 40% BPD 18% mood	43	56%	54%	9%
Cook 2011 [45]	Ohio *USA*	555	20% SS 26% BPD, 24% MDD	46	66%	37%	15%
Craig 2004 [38]	London *UK*	45	100% SS	38	33%	N/R	N/R
Davidson 2004 [41]	Connecticut *USA*	260	50% SS 34% mood	42	57%	18%	20%
Proudfoot 2012 [37]	New South Wales *Australia*	407	100% BPD	N/R	70%	N/R	N/R
Rivera 2007 [48]	New York *USA*	255	48% SS 26% BPD 22% MDD	38	49%	71%	N/R
Simon 2011 [27]	*USA*	118	100% BPD	N/R	72%	19%	N/R
Sledge 2011 [44]	Connecticut *USA*	89	69% SS 31% mood	41	49%	N/R	N/R
Van Gestel-Timmermans 2012 [50]	*Netherlands*	333	33% SS	44	66%	N/R	N/R
*Peer Delivered Services*							
Sells 2006 [43]	Connecticut *USA*	137	61% SS 70% multiple	42	39%	11%	N/R
Clarke 2000 [40]	Oregon *USA*	178	60% SS31% SM	37	39%	18%	N/R
Solomon 1995 [28]	Pennsylvania *USA*	96	82% SS 12% mood	41	47%	37%	N/R

^Φ^Participants with a schizophrenia spectrum (SS) disorder, bipolar disorder (BPD), major depressive disorder (MDD), substance misuse (SM), or mood disorder.

^Ω^Currently employed.

ΨTwo intervention groups (a peer support email list and an online bulletin board) were merged for analysis.

αRandomised in six teams.

Participants varied across trials in diagnoses. Two studies included only participants with bipolar disorder [27,37] and one study included only participants with schizophrenia spectrum disorders [38]. Of the remaining 15 studies with mixed populations of secondary mental health service users, seven involved a majority of participants diagnosed with schizophrenia [28,39-44], six involved a majority with mood disorders [45-50], and two failed to report data concerning participant diagnosis although all were mental health service users [36,51].

Trials also varied regarding participants in current employment (4% to 64%), and with comorbid substance misuse (0% to 72%). The median of the mean age was 42 years, and the median trial included 54% female participants. Interventions lasted between 3 weeks and 2 years. Four studies of peer support interventions reported follow-up data between 3-6 months beyond the end of treatment.

Reported details of the organisation and content of programmes from included studies are reported in Additional file 1. Trials of mutual support included peer-support groups (3) and an unmoderated internet support group (1). Two of the mutual support programmes involving peer support groups included additional access to drop-in centres. One involved access to advice for the project participants from a qualified clinician; no other training or supervision was reported for studies of mutual support interventions. In one trial, two eligible intervention groups were combined for analysis.

Trials of peer-support interventions included manualised programmes to improve self-management skills (6), less structured support including befriending, advocacy and help with social or practical problems (4), or a mixture of both (1). Two of the trials evaluated online programmes [27,37]. Arrangements for training and supervision for peer programme-providers varied (Additional file 1). In all studies of peer support interventions, support from peers was provided as an addition to standard care within mental health services.

Three trials of peer-delivered mental health services employed service users as case managers within Assertive Community Treatment or Intensive Case Management services (Additional file 1). Initial training was provided to peer-workers in all three studies, but the extent of this was not reported; supervision from clinical staff was reported as being provided.

### Risk of bias

**Figure 2:**
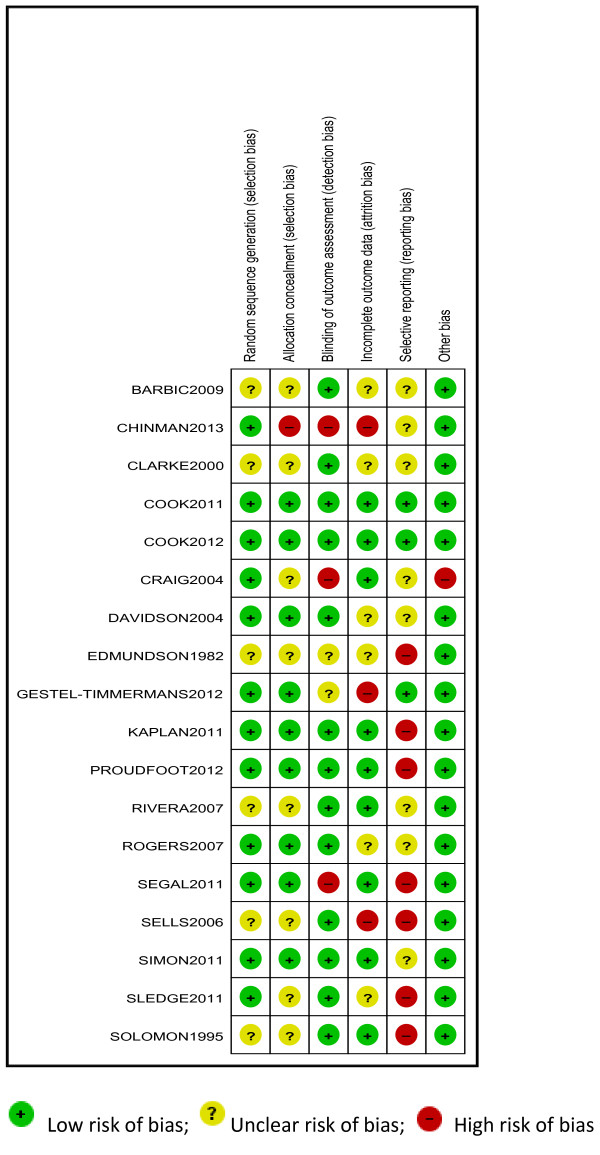
Risk of bias in included studies.

Using the Cochrane Risk of Bias Tool [52], sequence generation was not sufficiently described in 6 trials and concealment of the allocation sequence was not sufficiently described in 8 trials (Figure 2). Lack of blinding of assessors created a high risk of bias in 3 studies, and in 2 trials it was unclear if assessors were blind. At the trial level, 3 were at high risk of bias for missing data (i.e. attrition bias) and 6 were unclear. We were able to confirm by contacting trial authors and checking review protocols that 4 studies were completely free of selective outcome reporting (i.e. clearly reported all outcomes measured). Other included studies may have measured but not reported outcomes that are included in this review, and there may be unpublished trials of peer support, so results have been downgraded for risk of reporting bias. It was not clear if SDs for the cluster randomised trial were adjusted for clustering, and the precision of these effects (and their weight in the analysis) may be overestimated.

### Quantitative data synthesis

#### ***Post-treatment***

Results of the quantitative data synthesis of outcomes post-treatment are presented in Table 2.

**Table 2:** Summary of pooled effects at post-treatment

**Outcome**	***Trials***	***Participants***	***Effect size (95% CI)***	***Heterogeneity: I***^***2***^ ***; *** **Chi**^**2 **^ ***(p value)***	***Follow-up (months)***	***GRADE confidence rating***
*Mutual Support*	*4*	*2369*				
Hospitalisation [51]	1	80	RR = 0.50 (0.23, 1.11)	N/A	10	Very low^1,4,5^
Symptoms of psychosis	0	N/A	N/A	N/A	N/A	N/A
Employment	0	N/A	N/A	N/A	N/A	N/A
Quality of life [47]	1	300	SMD = -1.42 (-1.69, – 1.16)	N/A	12	Very low^1,4,5^
Overall psychiatric symptoms [49]	1	162	N/A “no difference in change”	N/A	N/A	N/A
Depression and anxiety [47]	1	300	SMD = -0.42 (-0.66, – 0.18)	N/A	12	Very low^1,4,5^
Recovery [47]	1	300	SMD = -0.11 (-0.35, 0.13)	N/A	12	Very low^1,4,5^
Hope [49]	1	162	N/A “no difference in change”	N/A	N/A	N/A
Empowerment [42,47,49]	3	2266	SMD = -1.44 (-2.79, – 0.09)	99%; 186.26 (p < 0.001)	8-12	Very low^1,2,4,5^
Satisfaction	0	N/A	N/A	N/A	N/A	N/A
*Peer-support*	*11*	*2805*				
Hospitalisation [38]	1	45	RR = 1.07 (0.55, 2.07)	N/A	9-12	Very low^1,4,5^
Duration of admission [38,44,48]	3	255	SMD = -0.22 (-0.72, 0.28)	72%; 7.16 (p = 0.03)		Very low^1,2,4,5^
Symptoms of psychosis [36,45]	2	696	SMD = -0.08 (-0.27, 0.03)	N/A	2-12	Low^4,5^
Employment	0	N/A	N/A	N/A	N/A	N/A
Quality of life [36,39,45,48,50]	5	1039	SMD = 0.04 (-0.16, 0.24)	52%; 8.38 (p = 0.08)	3-12	Very low^1,4,5^
Depression and anxiety [36,41,45]	3	861	SMD = -0.10 (-0.24, 0.03)	0%; 1.97 (p = 0.37)	2-12	Low^4,5^
Overall psychiatric symptoms [41,45,48]	3	753	SMD = -0.07 (-0.39, 0.24)	74%; 7.83 (p = 0.02)	3-9	Very low ^1,2,4,5^
Recovery [36,39,45,46]	4	1066	SMD = -0.24 (-0.39, – 0.09)	27%; 4.09 (p = 0.25)	2-12	Low^4,5^
Hope (SMD) [39,45,46,50]	4	1072	SMD = -0.14 (-0.27, -0.02)	7%; 3.21 (p = 0.36)	2-3	Very low ^1,4,5^
Empowerment [39,50]	2	286	SMD = -2.67 (-7.35, 2.02)	97%; 38.87 (p < 0.001)	3	Very low^1,2*,4,5^
Satisfaction [38,41,48]	3	332	SMD = 0.02 (-0.20, 0.23)	0%; 0.95 (p = 0.62)	9-12	Very low^1,4,5^
*Peer delivered services*	*3*	*411*				
Hospitalisation [40]	1^Ψ^	114	RR = 0.68 [0.45, 1.03]	N/A	24	Very Low^1,4,5^
Symptoms of psychosis	0	N/A	N/A	N/A	N/A	N/A
Employment [28]	1	96	N/A *“no differences”*	*N/A*	*N/A*	*N/A*
Quality of life (SMD) [28]	1	96	N/A *“no differences”*	*N/A*	*N/A*	*N/A*
Depression and anxiety	0	N/A	N/A	N/A	N/A	N/A
Overall psychiatric symptoms	0	N/A	N/A	N/A	N/A	N/A
Recovery	0	N/A	N/A	N/A	N/A	N/A
Hope	0	N/A	N/A	N/A	N/A	N/A
Empowerment	0	N/A	N/A	N/A	N/A	N/A
Satisfaction [28]	1	87	SMD = 0.48 (0.05, 0.91)	N/A	12	Very low^1,4,5^

This table shows the number of trials and participants assigned for each comparison. For each outcome, it lists the number of trials and participants included in the analysis.

Reasons for downgrade: ^1^Risk of bias, ^2^Inconsistency, ^3^Indirectness, ^4^Imprecision, ^5^Publication/Reporting Bias.

*RR* Relative Risk, *SMD* Standardised Mean Difference.

Ψ Solomon [28] measured the outcome and reported no difference.

All four studies of mutual support reported data that could be converted to standardised effects. No studies reported measures of symptoms of psychosis, employment, or user satisfaction that could be analysed. There was evidence of a non-significant effect from one study on hospitalisation (RR = 0.50, 0.23 to 1.11) and of a large significant effect on quality of life (SMD = -1.42, -1.69 to -1.16), but confidence in this evidence was graded very low using the GRADE system [26]. One study found no significant difference in psychiatric symptoms, but no data were provided [49]. Very low-graded evidence from another study suggested mutual support had a medium effect on symptoms of depression and anxiety (SMD = -0.42, -0.66 to -0.18) but no effect on recovery (SMD = -0.11, -0.35 to 0.13). One study reported no significant difference on hope, but no data were provided [49]. There was a very large effect on empowerment when three studies were combined (SMD = -1.44, -2.79 to -0.09), but the evidence was very low-graded and heterogeneity was considerable (I^2^ = 99%; Chi^2^ = 186.26, p < 0.00001); one study reported an extremely large effect [49] whilst the others reported no difference.

Of the eleven studies of peer-support, nine reported post-treatment data that could be converted to standardised effects. No studies reported employment outcomes. Very low-graded evidence found no beneficial effect of peer support on hospitalisation outcomes with one study reporting no effect on the number of people hospitalised (RR = 1.07, 0.55 to 2.07) and three studies reporting no effect on the duration of admission (SMD = -0.22, -0.72 to 0.28) with substantial heterogeneity (I^2^ = 72%; Chi^2^ = 7.16, p = 0.03). Low-graded evidence from two studies found that peer support had no effect on symptoms of psychosis (SMD = -0.08, -0.27 to 0.03). No significant effect on quality of life was reported in five studies (SMD = 0.04, -0.16 to 0.24; I^2^ = 52%; Chi^2^ = 8.38, p = 0.08) but the evidence was graded very low. Three studies reported very low-graded evidence showing no difference in psychiatric symptoms (SMD = -0.07, -0.39 to 0.24), but the studies were inconsistent (I^2^ = 74%; Chi^2^ = 7.83, p = 0.02). No significant effect of peer support was found on symptoms of depression and anxiety (SMD = -0.10, -0.24 to 0.03; I^2^ = 0%; Chi^2^ = 1.97, p = 0.37). Four studies did report a small positive effect on recovery (SMD = -0.24, -0.39 to -0.09), but there was moderate heterogeneity (I^2^ = 27%; Chi^2^ = 4.09, p = 0.25) and the evidence was graded low. There was very low-graded evidence in four studies of a small positive effect on hope (SMD = -0.14, -0.27 to -0.02; I^2^ = 7%, Chi^2^ = 3.21, p = 0.36). Two studies reported no difference in empowerment (SMD = -2.67, -7.35 to 2.02); one trial contains a discrepancy in description of the measure and the data presented [39], and there was considerable heterogeneity (I^2^ = 97%, Chi^2^ = 38.87, p < 0.001). Three studies reported very low-graded evidence showing no difference in user satisfaction (SMD = 0.02, -0.20 to 0.23; I^2^ = 0%; Chi^2^ = 0.95, p = 0.62).

A sensitivity analysis was conducted which excluded the Chinman (2013) cluster randomised trial [36], because of concerns that the low take-up of the trial intervention among service users in the intervention group services might have affected results. Results from the sensitivity analysis were not significantly different from those in the main analyses.

Of the four studies of peer-delivered services, two reported data that could be converted to standardised effects, but neither study reported measures of symptoms of psychosis, quality of life, overall psychiatric symptoms, depression or anxiety, recovery, hope, or empowerment that could be analysed. There was very low-graded evidence from one study of no significant difference in hospitalisation (RR = 0.68, 0.45 to 1.03). The second study reported a small negative effect for satisfaction (SMD = 0.48, 0.05 to 0.91), but the evidence was also graded very low. One study measured employment, quality of life, and hospitalisation but the study did not report data in a format that could be added to the analysis; the paper only reports significant differences [28].

#### ***Follow-up***

No studies of either mutual support or peer-delivered services reported follow-up data for any outcome. Four studies of peer-support reported follow-up data that could be converted to standardised effects. However, no peer support studies reported hospitalisation, employment or satisfaction outcomes after the post-treatment assessment (Table 3).

**Table 3:** Summary of pooled effects at follow-up

**Outcome**	***Trials***	***Participants***	***Effect size (95% CI)***	***Heterogeneity: I***^***2***^ ***; *** **Chi**^**2 **^ ***(p value)***	***Follow-up (months post-treatment )***	***GRADE confidence rating***
*Peer support*	4	1723				
Hospitalisation	0	N/A	N/A	N/A	N/A	N/A
Symptoms of psychosis [45]	1	448	SMD = -0.00 (-0.19, 0.18)	N/A	6	Low ^4,5^
Employment	0	N/A	N/A	N/A	N/A	N/A
Quality of life [45,50]	2	639	SMD = -0.24 (-0.40, -0.08)	0%; 0.00 (p = 0.98)	3-6	Very low^1, 4, 5^
Depression and anxiety [37,45]	2	721	SMD = -0.17 (-0.32, -0.03)	0%; 0.15 (p = 0.70)	6	Low^4,5^
Overall psychiatric symptoms [45]	1	448	SMD = -0.08 (-0.26, 0.11)	N/A	6	Low^4,5^
Recovery [45,46]	2	757	SMD = -0.23 (-0.37, -0.09)	0%, 0.71 (p = 0.40)	6	Low^4,5^
Hope [45,46,50]	3	967	SMD = -0.24 (-0.46, -0.02)	65%; 5.74 (p = 0.06)	3-6	Very low^1,2,4,5^
Empowerment [46,50]	2	538	SMD = -0.25 (-0.43, -0.07)	12%; 1.13 (p = 0.29)	6	Very low^1,4,5^
Satisfaction	0	N/A	N/A	N/A	N/A	N/A

Reasons for downgrade: ^1^Risk of bias, ^2^Inconsistency, ^3^Indirectness, ^4^Imprecision, ^5^Publication/Reporting Bias.

*RR* Relative Risk, *SMD* Standardised Mean Difference.

There was low-graded evidence from one study of no effect of peer support at six month follow-up on symptoms of psychosis (SMD = -0.03, -0.22 to 0.16) and no effect on overall psychiatric symptoms (SMD = -0.08, -0.26 to 0.11). Combining the studies, there was very low-graded evidence of no effect on quality of life (SMD = -0.24, -0.40 to -0.08; I^2^ = 0%; Chi^2^ = 0.00, p = 0.98) at three and six month follow-up. There was low-graded evidence of a small positive effect on both symptoms of depression (SMD = -0.17, -0.32 to -0.03; I^2^ = 0%; Chi2 = 0.15, p = 0.70) and on recovery (SMD = -0.23, -0.37 to -0.09; I^2^ = 0%; Chi^2^ = 0.71, p = 0.40). A small positive effect was also reported for hope (SMD = -0.24, -0.46 to -0.02) at three and six month follow-up by three studies, although the evidence was graded very low and heterogeneity was substantial (I^2^ = 65%; Chi^2^ = 5.74, p = 0.06). Two studies of peer support reported a small positive effect on empowerment (SMD = -0.25, -0.43 to -0.07; I^2^ = 12%; Chi^2^ = 1.13, p = 0.29) at six month follow-up but confidence in the evidence was graded as very low.

## Discussion

### Main findings

This review provides a current account of research about a widely and increasingly used intervention. Peer delivered interventions are increasingly common in voluntary settings, and as part of secondary mental health care provision. However the findings of this review suggest there is little current evidence regarding the effectiveness of peer support for people with serious mental illness. Due to variation between trials in outcomes assessed, analyses for all outcomes included data from one or only a few trials, limiting confidence in the generalizability of results. All but two studies exhibited some serious risk of bias, and confidence in the evidence for all outcomes at study endpoint was low or very low. Studies of mutual or uni-directional peer support, where analyses could be carried out, provide little evidence that peer support improves service users’ hospitalisation, overall symptoms or satisfaction outcomes as an addition to treatment as usual. There were some positive results for outcomes specifically relating to a recovery process, i.e. self-rated recovery, hope and empowerment [53]. These may reflect changes in areas most directly addressed by peer support, but these outcomes were not consistent and could be explained by reporting bias. Analyses from trials of mutual support programmes found (with heterogeneity between studies for some outcomes) a positive effect for empowerment but not for hope or recovery; analyses from trials of peer support services found positive effects for recovery and hope but not for empowerment. Three American studies found few differences in outcomes between peer and clinician-provided case management; however these studies were not designed or powered as non-inferiority trials, so the results cannot be interpreted as evidence of equivalence.

### Strengths and limitations

Unlike previous reviews, this review formally compares evidence from randomised trials about the efficacy of all community-based peer support programmes designed to help with recovery from severe mental illness. Searches were highly sensitive, and the review provides a comprehensive synthesis of important outcomes. Main outcomes were identified a priori, but we added two outcomes (hope and empowerment) post-hoc. These were commonly reported in included studies and considered important by triallists and service users.

In line with best practice guidelines for systematic reviews [54], we have not sought to supplement existing randomised trial evidence by including non-randomised trials in our review [55]. Randomised trials provide the best evidence of efficacy, and including other trial designs could have led to misleading results; however, our conclusions are consequently limited by the small number of studies included in analyses. Conclusions are further limited by variation in programme content and trial populations, and the low confidence in available evidence due to poor study design and incomplete reporting. In the absence of clearly specified models or fidelity criteria for peer support, we categorised peer support according to previous typologies. Nonetheless, there was substantial variation in programme content and participant characteristics. These factors may have contributed to observed heterogeneity; i.e., outcomes were inconsistent in magnitude and direction both within and across analyses. There were not enough studies to conduct further sub-group analyses. Current evidence is insufficient to conclude that peer support interventions are ineffective, but also insufficient to recommend peer support in general or any particular type of peer intervention. It is equally unclear if there are any critical ingredients that might contribute to programme success or appropriate target populations.

### Comparison with earlier reviews

Previous systematic reviews acknowledge the limitations of the evidence and call for further good quality randomised trials [20,21,24]. However, they often accentuate positive findings from poor quality evidence, highlighting “limited but promising evidence,” [20] the “potential to drive through recovery-focused changes in services,” [21] and the possibility for peer-provided mental health services “without detrimental effect” [24] or “with no evidence of harm” [22].

Previous reviews may include more positive appraisals for several reasons. Many seem to assume that lack of significant differences between active interventions demonstrates equivalence, which is inappropriate in trials designed to test for superiority that are neither designed nor powered to demonstrate non-inferiority. Additionally, some reviews include non-randomised studies, the results of which may differ from randomised trials as a result of more inclusive selection criteria or selection bias. A previous review, for example, reported that the majority of non-RCT evidence suggests peer support had a positive effect on admission rates [22]. Finally, narrative reviews and narrative syntheses do not account for differences in precision and statistical heterogeneity among studies, and lack of formal comparisons can obscure the effects of selective outcome reporting. This review demonstrates that greater caution is warranted because available studies do not provide empirical evidence for the effectiveness of peer support.

### Implications for future research

Further research is needed to develop and test theory-based interventions and to describe them clearly. Peer support programmes may not follow highly specified theoretical models, and they may not have well-defined goals. We attempted to distinguish different types of peer support using an existing typology, but programmes varied in content, target client group, group or individual delivery, face-to-face or internet-based delivery, degree of support from local mental health services, and extent of provider training. Among the studies of peer-delivered services included in our review, some were comparatively brief, structured self-management focused programmes [27,37,39,45,46,50] and others were longer-term and less structured [38,41,44,48]. This distinction might be helpfully reflected in future typologies. Developing and describing clear models of peer support would facilitate future trials and help future reviewers synthesise them more effectively. Trials should as far as possible include a process evaluation, to help understand whether the peer support programme was implemented as intended and mechanisms by which any effects of this complex intervention are achieved [56].

With a few exceptions [45,46], included studies were poorly reported and failed to adhere to the CONSORT guidelines for reporting trials [57]. Reporting guidelines may be insufficient for studies of complex interventions [58], but most included studies omitted basic details about their methods and outcomes [59]. These limitations are common in studies of psychological interventions [60], and this review reinforces the need to improve standards for reporting trials of social and psychological interventions. Future trials of peer-delivered interventions should be registered in advance [61], publish their protocols, clearly state all outcomes and time points to be assessed, describe the interventions provided, and report outcomes in full.

### Implications for policy and practice

In response to widespread advocacy from a range of stakeholder groups, peer support programmes have been implemented internationally [9,11,62-64]. Peer support has been positively appraised in qualitative literature as beneficial for service user recipients [65,66] and as a mechanism for challenging attitudes of clinical staff and contributing to culture change within mental health services [67]. However, the lack of empirical evidence regarding its effectiveness does not support a recommendation or mandatory requirement from policymakers for mental health services to provide peer support programmes. If peer support programmes are identified as desirable and implemented locally, service planners should include rigorous evaluations to determine if these programmes affect outcomes of interest. Where possible, given current knowledge, implementation of peer-provided support programmes should be organised in the context of a formal research study.

## Conclusion

Despite the promotion and uptake of peer support internationally, there is little evidence from current trials about the effectiveness of peer support for people with severe mental illness. Although there are few positive findings, this review has important implications for policy and practice: current evidence does not support recommendations or mandatory requirements from policy makers for mental health services to provide peer support programmes.

## Competing interests

All authors declare: no support from any other organization for the submitted work; no financial relationships with any organizations that might have an interest in the submitted work in the previous 3 years; no other relationships or activities that could appear to have influenced the submitted work.

## Authors’ contributions

BLE contributed to study design, data collection, analysis and interpretation of data and writing up the paper. EMW contributed to study design, data collection, analysis and interpretation of data and writing up the paper. BH contributed to study design, data collection, analysis and interpretation of data and writing up the paper. HI contributed to data collection and writing up the paper. EB contributed to data collection and writing up the paper. SP contributed to study design and writing up the paper. SJ contributed to study design, interpretation of data and writing up the paper. TK contributed to study design and writing up the paper. All authors read and approved the final manuscript.

## Pre-publication history

The pre-publication history for this paper can be accessed here:

http://www.biomedcentral.com/1471-244X/14/39/prepub

## Supplementary Material

Additional file 1 **Peer support for serious mental illness. Appendix 1.** Review electronic search terms. OVID Search Strategy (Medline, PsycINFO, Embase). Peer Support for Serious Mental Illness: **Appendix 2.** Characteristics of interventions from included studies. Click here for file
