# Supplementary material for: A systematic review and meta-analysis of randomised controlled trials of peer support for people with severe mental illness
Source: BMC Psychiatry. 2014 Feb 14;14:39. doi: 10.1186/1471-244X-14-39 (PMC3933205; doi:10.1186/1471-244X-14-39)
Supplement: Additional file 1 — Peer support for serious mental illness. Appendix 1. Review electronic search terms. OVID Search Strategy (Medline, PsycINFO, Embase). Peer Support for Serious Mental Illness: Appendix 2. Characteristics of interventions from included studies. [file 1471-244X-14-39-S1.docx]

**Peer Support for Serious Mental Illness: Appendix 1: Review electronic search terms**

**OVID Search Strategy (Medline, PsycINFO, Embase)**

| 1 | "explode schizophrenia"/ or (psychosis$ or psychotic$).hw. |
| --- | --- |
| 2 | 1 use emez |
| 3 | paranoid disorders/ or exp psychotic disorders/ or exp schizophrenia/ or "schizophrenia and disorders with psychotic features"/ |
| 4 | 3 use mesz, prem |
| 5 | exp psychosis/ or exp schizophrenia/ |
| 6 | 5 use psyh |
| 7 | ((chronic$ or serious$ or sever$) adj2 mental$ adj2 (ill$ or disorder$)).ti,ab,hw,id. or (delusional disorder$ or hebephreni$ or oligophreni$ or psychoses or psychosis or psychotic$ or schizo$).ti,ab,id. |
| 8 | akathisia/ or dyskinesia/ or neuroleptic malignant syndrome/ |
| 9 | 8 use emez |
| 10 | akathisia, drug-induced/ or dyskinesias/ or dyskinesia, drug-induced/ or neuroleptic malignant syndrome/ |
| 11 | 10 use mesz, prem |
| 12 | akathisia/ or exp dyskinesia/ or neuroleptic malignant syndrome/ |
| 13 | 12 use psyh |
| 14 | (akathisi$ or acathisi$ or (neuroleptic$ and ((malignant and syndrome) or (movement adj2 disorder))) or (tardiv$ and dyskine$)).ti,ab,id. or ((parkinsoni$ or neuroleptic induc$).ti,ab,id. not (parkinson$ and disease).ti.) |
| 15 | or/2,4,6-7,9,11,13-14 |
| 16 | friend/ or home care/ or home rehabilitation/ or peer counseling/ or peer group/ or psychosocial care/ or social network/ or social support/ or social worker/ or support group/ or vocational rehabilitation/ or voluntary worker/ or volunteer/ |
| 17 | 16 use emez |
| 18 | community networks/ or education, nonprofessional/ or friends/ or home care services/ or hotlines/ or peer group/ or rehabilitation, vocational/ or self-help groups/ or social support/ or social work/ or social work, psychiatric/ or voluntary workers/ |
| 19 | 18 use mesz, prem |
| 20 | friendship/ or home care/ or home visiting programs/ or hot line services/ or peer counseling/ or peer relations/ or peers/ or exp psychosocial rehabilitation/ or social casework/ or social group work/ or social support/ or exp social networks/ or social workers/ or exp support groups/ or vocational counselors/ or volunteers/ |
| 21 | 20 use psyh |
| 22 | (befriend$ or be$1 friend$ or buddy or buddies or ((community or lay or paid or support) adj (person or worker$)) or ((community$ or home) adj (based or visit$)) or ((consumer$ or friend$ or lay or mutual$ or peer$ or social$ or volunteer$) adj3 (help$ or network$ or support$ or visit$)) or ((consumer$ or peer$ or social$ or support$ or volunteer$) adj2 (group$ or network$)) or ((consumer$ or friend$ or lay$ or peer$ or user$ or volunteer$) adj (based or counsel$ or deliver$ or interact$ or led or mediat$ or operated or provides or provider$ or run$)) or ((consumer$ or friend$ or lay$ or peer$ or relation$ or support$) adj3 trust$) or (coping adj3 (behavio?r$ or skill$)) or (emotion$ adj (focus$ or friend$ or relation$)) or ((dyadic or loneliness or psychosocial$ or psycho social$) adj2 (intervention$ or program$ or therap$ or treat$)) or ((emotion$ or one to$1 one or transition$) adj support$) or (lay adj (led or run)) or ((lay or peer) adj5 (advisor$ or consultant or educator$ or expert$ or facilitator$ or instructor$ or leader$ or person$ or tutor$ or worker$)) or expert patient$ or mutual aid or (peer$ adj3 (advic$ or advis$ or counsel$ or educat$ or mentor$)) or ((social or psychosocial or psycho social) adj (adapt$ or support$)) or supportive relationship$ or social interaction program$ or support$ listening or recover inc or schizophrenics anonymous or visit$ service$ or (voluntary adj3 worker$) or (volunteer$ adj5 (trained or aide))).ti,ab. |
| 23 | (helpline or help line or ((phone$ or telephone$) adj3 (help$ or instruct$ or interact$ or interven$ or mediat$ or program$ or rehab$ or strateg$ or support$ or teach$ or therap$ or train$ or treat$ or workshop$)) or ((phone or telephone$) adj2 (assist$ or based or driven or led or mediat$))).ti,ab. |
| 24 | or/17,19,21-23 |
| 25 | exp "clinical trial (topic)"/ or exp clinical trial/ or crossover procedure/ or double blind procedure/ or placebo/ or randomization/ or random sample/ or single blind procedure/ |
| 26 | 25 use emez |
| 27 | exp clinical trial/ or cross-over studies/ or double-blind method/ or placebos/ or random allocation/ or "randomized controlled trials as topic"/ or single-blind method/ |
| 28 | 27 use mesz, prem |
| 29 | (clinical trials or placebo or random sampling).sh,id. |
| 30 | 29 use psyh |
| 31 | (clinical adj2 trial$).ti,ab. |
| 32 | (crossover or cross over).ti,ab. |
| 33 | (((single$ or doubl$ or trebl$ or tripl$) adj2 blind$) or mask$ or dummy or doubleblind$ or singleblind$ or trebleblind$ or tripleblind$).ti,ab. |
| 34 | (placebo$ or random$).ti,ab. |
| 35 | treatment outcome$.md. use psyh |
| 36 | animals/ not human$.mp. use emez |
| 37 | animal$/ not human$/ use mesz, prem |
| 38 | (animal not human).po. use psyh |
| 39 | (or/26,28,30-35) not (or/36-38) |
| 40 | 15 and 24 and 39 |

### Peer Support for Serious Mental Illness: Appendix 2: Characteristics of interventions from included studies

| Study | Comparison | Brief description of trial intervention |
| --- | --- | --- |
| Edmundson 1982 [^51^](#_ENREF_51) | MS: Community Network Development (CND)  TAU: Outpatient Care (CMHC) | Project attached to a community mental health service. Group + 1:1 support with social activities and accessing services. Ongoing support over 10 month study period. Access to support and advice from a qualified clinician. |
| Kaplan 2011 [^47^](#_ENREF_47) | MS: Internet peer support email list  MS Bulletin board  TAU: Wait-list | Internet peer support: bulletin board and chat room. Ongoing over 12 month study period. |
| Rogers 2007 [^42^](#_ENREF_42) | MS: Consumer operated service programs (COSP) +TAU  TAU: Outpatient services | Project attached to a community mental health service. Social drop-ins, group meetings and information about illness and services. Ongoing support over 12 month study period. |
| Segal 2011 [^49^](#_ENREF_49) | MS: Consumer operated service programs (COSP) +TAU  TAU: Outpatient services | As above - 8 month study period. |
| Barbic 2009 [^39^](#_ENREF_39) | PS: Recovery Workbook + TAU  TAU: Professional-led ACT | Structured group programme developing coping strategies, building support and setting personal goals. 12 weekly meetings of 2 hours duration. Training and supervision of peer supporters was not specified. |
| Chinman 2013 [^36^](#_ENREF_36) | PS: 2 additional peer support workers employed as “floating” staff within each ACT team  TAU: Assertive community treatment team | Two peer specialists were employed in each ACT team. They did not act as case managers but offered additional “floating” support wherever needed, helping with case management activities, developing recovery plans and delivering an Illness Management and Recovery programme, over 1 year study period. Peer providers received 30 hrs initial peer support training + 2 days IMR training, then weekly supervision from clinical staff. |
| Cook 2011[^45^](#_ENREF_45) | PS: Wellness Recovery Action Plan (WRAP) + TAU  TAU: Case management (70%) | Wellness Recovery Action Plan (WRAP)  Structured group programme developing a wellness maintenance plan and a crisis plan. 8 weekly meetings of 2.5 hours duration.  Peer supporters undertook formal WRAP trainer accreditation and 2.5 days’ study-specific training. A “back-up” clinician was available for support and advice following all meetings. |
| Cook 2012[^46^](#_ENREF_46) | PS: Building Recovery of Individual Dreams and Goals through Education and Support (BRIDGES) + TAU  TAU: Community mental health services | Building Recovery of Individual Goals and Dreams through Education and Support (BRIDGES): structured group programme developing problem solving strategies, building support, providing information about illness and medication, relapse prevention planning. 8 weekly meetings of 2.5 hours duration.  Peer supporters undertook formal BRIDGES trainer accreditation and ongoing feedback from observation by study coordinators. |
| Craig 2004 [^38^](#_ENREF_38) | PS: Peer support + TAU  TAU:Intensive Case Management | Peer workers in ACT* services  Peers offers social care, advocacy and befriending + recreational group activities additional to standard care; ongoing support over 1 year study period.  Peer supporters were given 6 weeks’ training and induction from ACT clinicians; details of supervision not reported. |
| Davidson 2004 [^41^](#_ENREF_41) | PS: The Partnership Project + TAU  TAU: Outpatient Care (CMHC)  Other: Non-peer partner (befriending) + TAU (Not analysed) | 1:1 and group social and leisure activities in community locations: ongoing support over 9 month study period (2-4 hours per week)  Peer supporters were given one initial training session and ongoing monthly supervision facilitated by clinical and peer staff. |
| Proudfoot 2012[^37^](#_ENREF_37) | PS: Informed supporters + psychoeducation programme  TAU : TAU + psychoeducation programme, no peer support  TAU: TAU but no psychoeducation or peer support | Bipolar Education Programme + Informed Supporters  8 session online psycho-education programme about bipolar disorder covering causes of illness, treatments, personal wellbeing plans and social support + additional email peer support during 8 week study programme  Training (unspecified) was provided by clinicians + monthly supervision. |
| Rivera 2007 [^48^](#_ENREF_48) | PS: Peer Support + TAU  TAU: Intensive Case management  Other TAU: Treated exclusively in clinic (Not analysed) | 1:1 and group support from peer workers in ICM** services, helping build social support and activities, additional to standard care. Ongoing support over 12 month study period.  Peer supporters received one week of initial training and weekly ongoing training and supervision from clinical staff. |
| Simon 2011[^27^](#_ENREF_27) | PS: Peer coaches + online psychoeducation + TAU  TAU: online psychoeducation + TAU, no peer support | “MyRecoveryPlan”+ peer coaching  Online recovery programme for people with bipolar disorder with assertive engagement, unlimited support and coaching provided online by peer coaches during 3-week intervention period  Peer supporters with 4 years’ previous experience in peer support roles were recruited; they undertook an accredited training course and had access to weekly teleconference supervision from clinical staff. |
| Sledge 2011 [^44^](#_ENREF_44) | PS: Peer mentor scheme + TAU  TAU: various community services | Untitled “peer companion” model intervention  1:1 unstructured support, content agreed collaboratively: ongoing over 12 month study period  Peer supporters received an initial training programme (unspecified) and weekly supervision from clinical staff. |
| Van Gestel-Timmermans 2012[^50^](#_ENREF_50) | PS: “Recovery is up to you” peer-facilitated self management + TAU  TAU: Participants used a range of mental health services | “Recovery is up to you”: 12 weekly 2-hour meetings for a group peer-facilitated programme promoting recovery, including goal-setting, social support and illness management  Peer supporters had completed the programme themselves and received additional training (details + any supervision not specified). |
| Sells 2006 [^43^](#_ENREF_43) | PDS: Peer-delivered case management (CM)  TAU: Case management | Peer-provided ACT: peer workers acting as ACT caseworkers, supported by a psychiatrist and a nurse consultant: ongoing support over 2 year study period including home visits and meetings at ACT services.  Peer providers received initial training and supervision from the ACT clinical team (duration/frequency not specified).  *“The overarching goal of didactic training was to provide peer staff with.. instruction of key areas of case management”* |
| Clarke 2000 [^40^](#_ENREF_40) | PDS: Consumer-provided ACT  TAU: Professional-led ACT  Other: Outpatient Care (CMHC) (Not analysed) | Peer-provided case management: peer workers acting as case managers with a recovery focus (i.e. identifying strengths and developing recovery skills): ongoing support over 2 year study duration, including home visits and meetings at ACT services  Peer providers received initial training and supervision from the clinical team (duration/frequency not specified).  *“An RCT comparing: a) an ACT program staffed by mental health consumers; b) an ACT program staffed by non-consumers”* |
| Solomon 1995 [^28^](#_ENREF_28) | PDS: Consumer-provided case management (CM)  TAU: Case management | Peer provided ICM: peer workers acting as ICM case managers: ongoing support over 2 year study duration (mean 1.6 hours direct contact per participant per week)  Peer-providers received initial training from a peer manager (duration not specified), then weekly supervision from a peer manager and access to advice from a psychiatrist in the ICM team.  *“An RCT of a team of case managers who are mental health consumers compared to a team of non-consumers”* |

* ACT = Assertive Community Treatment ** ICM = Intensive Case Management
